# A highlightedly improved method for isolating and characterizing calcium oxalate crystals from tubercles of *Mammillaria schumannii*

**DOI:** 10.1186/s13007-023-01110-1

**Published:** 2023-11-27

**Authors:** Changying Li, Chunli Chen, Lihong Qin, Dengyue Zheng, Qian Du, Qiandong Hou, Xiaopeng Wen

**Affiliations:** 1 College of Life Sciences, Key Laboratory of Plant Resource Conservation and Germplasm Innovation in Mountainous Region (Ministry of Education), Institute of Agro-Bioengineering, Guizhou University, Guiyang, 550025 Guizhou China; 2 College of Life Science and Technology, Huazhong Agricultural University, Wuhan, 430070 Hubei China

**Keywords:** Calcium oxalate (CaOx), Biomineral, CaOx crystal isolation methods, Organic matrix isolation and purification, SEM–EDS, Raman spectrum

## Abstract

**Background:**

Calcium oxalate (CaOx) is the most prevalent and widespread biomineral in plants and is involved in protective and/or defensive functions against abiotic stress factors. It is, however, expected that this function has an extremely significant contribution to growth processes in plants bearing large amounts of CaOx, such as cacti growing in desert environment.

**Results:**

In our research, small-sized CaOx crystals (≤ 20 µm) with tetrahedral or spherical shapes were observed to dominate in each epidermal and cortical cell from the tubercles of *Mammillaria schumannii*, a species from the Cereoideae subfamily, having tubercles (main photosynthetic organs) united with adjacent ones almost into ridges on its stem. Because they have potential significant functions, differential centrifugations after mechanical blending were used to obtain these small-sized CaOx crystals, which extremely tend to adhere to tissue or suspend in solution. And then the combined Scanning Electron Microscope Energy Dispersive System (SEM–EDS) and Raman spectroscopy were further performed to demonstrate that the extracted crystals were mainly CaC_2_O_4_·2H_2_O. Interestingly, spherical druses had 2 obvious abnormal Raman spectroscopy peaks of -CH and -OH at 2947 and 3290 cm^−1^, respectively, which may be attributed to the occluded organic matrix. The organic matrix was further extracted from spherical crystals, which could be polysaccharide, flavone, or lipid compounds on the basis of Raman spectroscopy bands at 2650, 2720, 2770, and 2958 cm^−1^.

**Conclusions:**

Here we used a highlightedly improved method to effectively isolate small-sized CaOx crystals dominating in the epidermal and cortical cells from tubercles of *Mammillaria schumannii*, which extremely tended to adhere plant tissues or suspend in isolation solution. And then we further clarified the organic matrix getting involved in the formation of CaOx crystals. This improved method for isolating and characterizing biomineral crystals can be helpful to understand how CaOx crystals in cacti function against harsh environments such as strong light, high and cold temperature, and aridity.

**Supplementary Information:**

The online version contains supplementary material available at 10.1186/s13007-023-01110-1.

## Background

Calcium oxalate (CaOx) is the most prevalent and widespread biomineral in plants [1, 2]. The variety of morphology features of CaOx crystals distributed in different organs of plants have been involved in protective and/or defensive functions against abiotic stress factors. Crystal cells are commonly found in the epidermal or parenchymatic tissue layers as well as adjacent veins, especially near the phloem [3]. The former usually has a function in the maintenance and photoprotection of the photosynthetic apparatus, and the latter aims to excrete the excessive calcium. [4, 5] CaOx crystals in plants are mainly whewellite (CaC_2_O_4_.H_2_O) and weddellite (CaC_2_O_4_.2H_2_O) [6]. Plants also make crystals of calcium oxalate in an intriguing variety of defined shapes [7]. The morphology of CaOx crystals in plants is mainly raphide, druse, prism, styloid, and crystal sand [8, 9].

Calcium oxalate (CaOx) forms within intravacuolar membrane chambers, termed crystal chambers, that differentiate and proliferate exclusively in the vacuoles of crystal cells [7]. The composition of intravacuolar membrane chambers in plants is clearly relevant to understanding how plant cells control crystal morphology. The chamber membrane could regulate crystal morphology by controlling the relative rate of transfer of Ca and oxalic acid into the crystallization space, and thus the ratio of these two ions, which can affect crystal morphology [10]. The ratio of these two ions exactly regulates the hydration state of CaOx, and there is a good correlation between hydration state and CaOx crystal shapes. It’s also conceivable that other vacuolar elements, such nucleating agents, have a role in regulating crystal growth and shape [11]. There is ample evidence for the crystal structure contains matrix components, which are noncrystalline substances [12–15]. Based on the research by Webb on biomacromolecules connected to crystals in grape (*Vitis*) leaves, the grape leaves contain a variety of glycoprotein types that compose the organic substrates. The result indicated that the substrates were what mostly affected the crystal morphology [16].

The structure and functions of CaOx crystals are tightly connected. The extremely large size of crystals such as styloids, which may span the entire cross section of a leaf, can be a structural deterrent against grazing [11]. Additionally, it was also demonstrated that CaOx with a special shape plays a direct role in light scattering, reducing the steep light gradient and thus enabling the leaf to use the incoming light flux more efficiently [17]. Moreover, the plant *Tragia ramosa* is covered with stinging hairs, which consist of an elongated stinging cell containing a large needle-shaped styloid crystal with a groove along one edge and a branched base [18]. When an animal or human brushes up against these hairs, the tip of the cell ruptures, allowing the needle-shaped crystal to puncture the dermis of the animal.

The identification of structural characteristics of CaOx crystals contributes to understanding their protective and/or defensive functions against abiotic stress factors. However, isolating of CaOx crystals from plant tissue is a challenge [19, 20]. The researchers used pectinase, cellulase, and density gradient centrifugation to separate and remove tissues from plants, as well as added the chemical reagents (e.g. isopropanol and Triton X-100) to isolate pure CaOx crystals [21–24], but none of their protocols evaluated whether they had isolated CaOx crystals in large and small sizes. The biggest difficulty is separating the crystals from the mixture of plant tissues. Some of the reported protocols may lead to crystal damage or omit the small-sized crystals [25, 26]. Moreover, further isolating and purifying the organic matrix from the separated CaOx crystals is a delicate and sophisticated procedure. Samples of high purity samples are necessary for chemical analysis and identification. Therefore, a nondestructive, simple, and effective methodology is genuinely needed for the isolation of CaOx crystals from living organisms.

For the protective and/or defensive function of CaOx crystals, which has an extremely significant contribution to growth processes in plants bearing large amounts of CaOx, such as cacti growing in desert environments [4], isolating and characterizing calcium oxalate crystals from cacti is particularly important. Here we developed a highlightedly improved method to effectively isolate and characterize small-sized CaOx crystals from tubercles of *Mammillaria schumannii*, a species from the Cereoideae subfamily having tubercles (main photosynthetic organs) united with adjacent ones almost into ridges on its stem. The highlightedly improved methodology in our research can help us comprehend how CaOx crystals in cacti function against harsh environments such as strong light, high and cold temperature, and aridity.

## Results

### The distribution of CaOx crystals in tubercles of *Mammillaria schumannii*

**Fig. 1:**
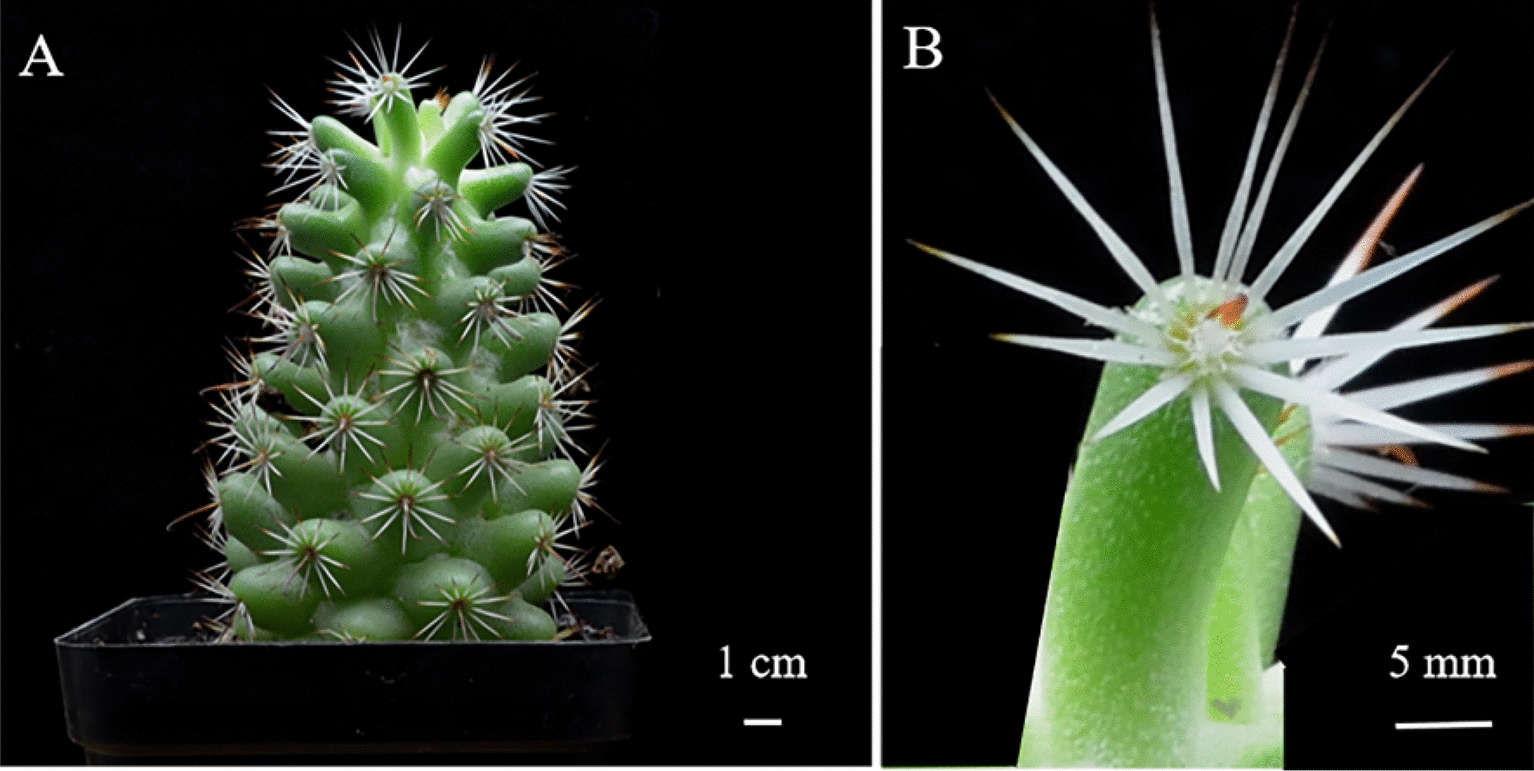
*Mammillaria schumannii* (**A**) and its tubercles (**B**)

**Fig. 2:**
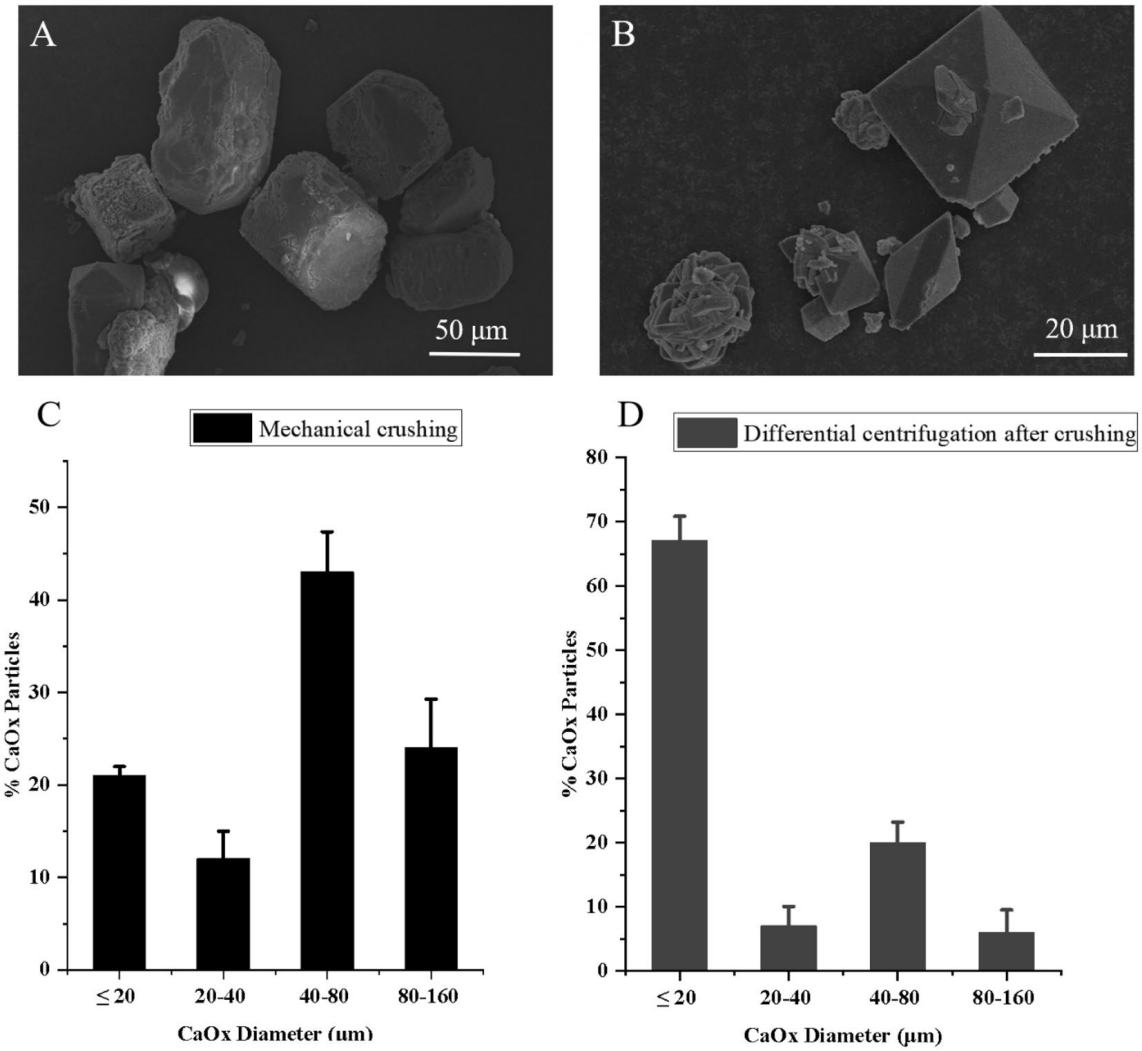
Scanning Electron Microscopy (SEM) images of CaOx crystals collected by mechanical crushing (**A**) and differential centrifugation after mechanical crushing (**B**), and size distributions of the extracted CaOx crystals(**C**, **D**)

According to polarized microscopy photographs of a cross section of the tubercle of *Mammillaria schumannii*, a significant amount of small-sized (≤ 20 µm) CaOx crystals were predominately distributed in epidermal and cortical (parenchymal) cells (Figs. 1, 2A and B). The morphologies of CaOx crystals in the cortex were tetrahedral or spherical (Fig. 2C and D). Additionally, only a small amount of CaOx crystals with a larger size (> 40 μm) were observed in vascular bundles (Additional file 1: Figure S1). It is necessary to isolate and clarify the structural properties of these massive, small-sized crystals in epidermal or cortical cells because they may have significant functions.

### The isolation of CaOx crystals from tubercles of *Mammillaria schumannii*

**Fig. 3:**
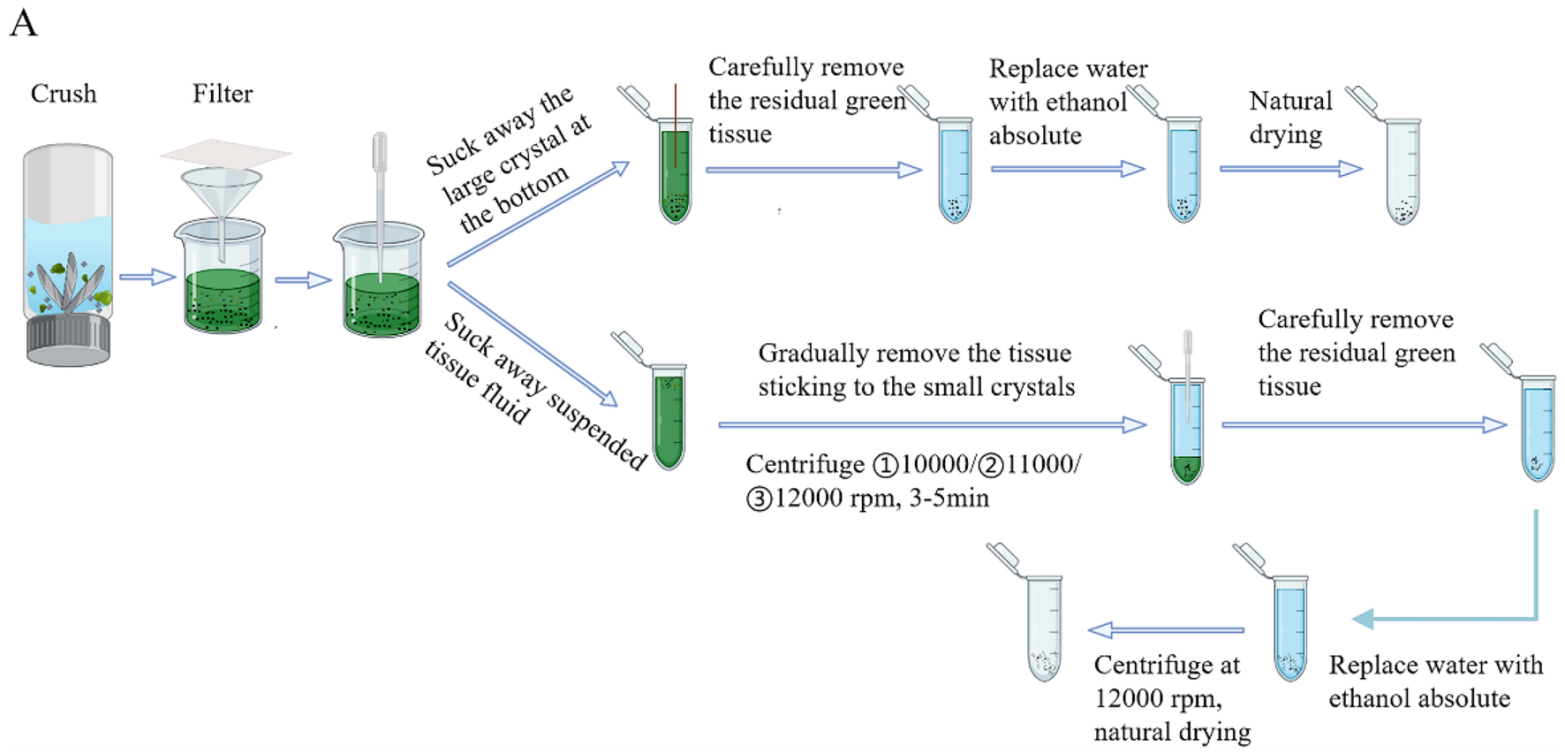
The method of collecting CaOx crystal through differential centrifugation after the mechanical blending. Blending the tubercles of Mammillaria schumannii, large-sized crystals settled down at the bottom of the blender. After collecting, three centrifugations of the blended solution were performed in order to separate the small-sized crystals from the plant tissues (**A**)

After manually blending the tubercles of *Mammillaria schumannii* (Fig. 1), large-sized crystals were immediately released, which can be easily collected at the bottom of the blender (Fig. 3 and Additional file 1: Fig. S2). After that, three centrifugations of the blended solution were performed in order to separate the small-sized crystals from the plant tissues (Fig. 3 and Additional file 1: Fig. S2). Centrifuged once at 10,000 rpm for 3–5 min, the supernatant was stirred to remove the bigger plant tissue with a toothpick; after the second centrifugation at 11,000 rpm for 3–5 min, the supernatant was again gently stirred to get rid of the resuspended tissue with a 200 μL pipette; the third time of centrifugation lasted for 3–5 min at 12,000 rpm, and the residual tissue had to be removed very carefully. It must be extremely cautious to avoid the loss of small-sized crystals during the process of getting rid of residual plant tissues.

### Size and morphological characteristics of crystals isolated by the differential centrifugation after mechanical blending.

**Fig. 4:**
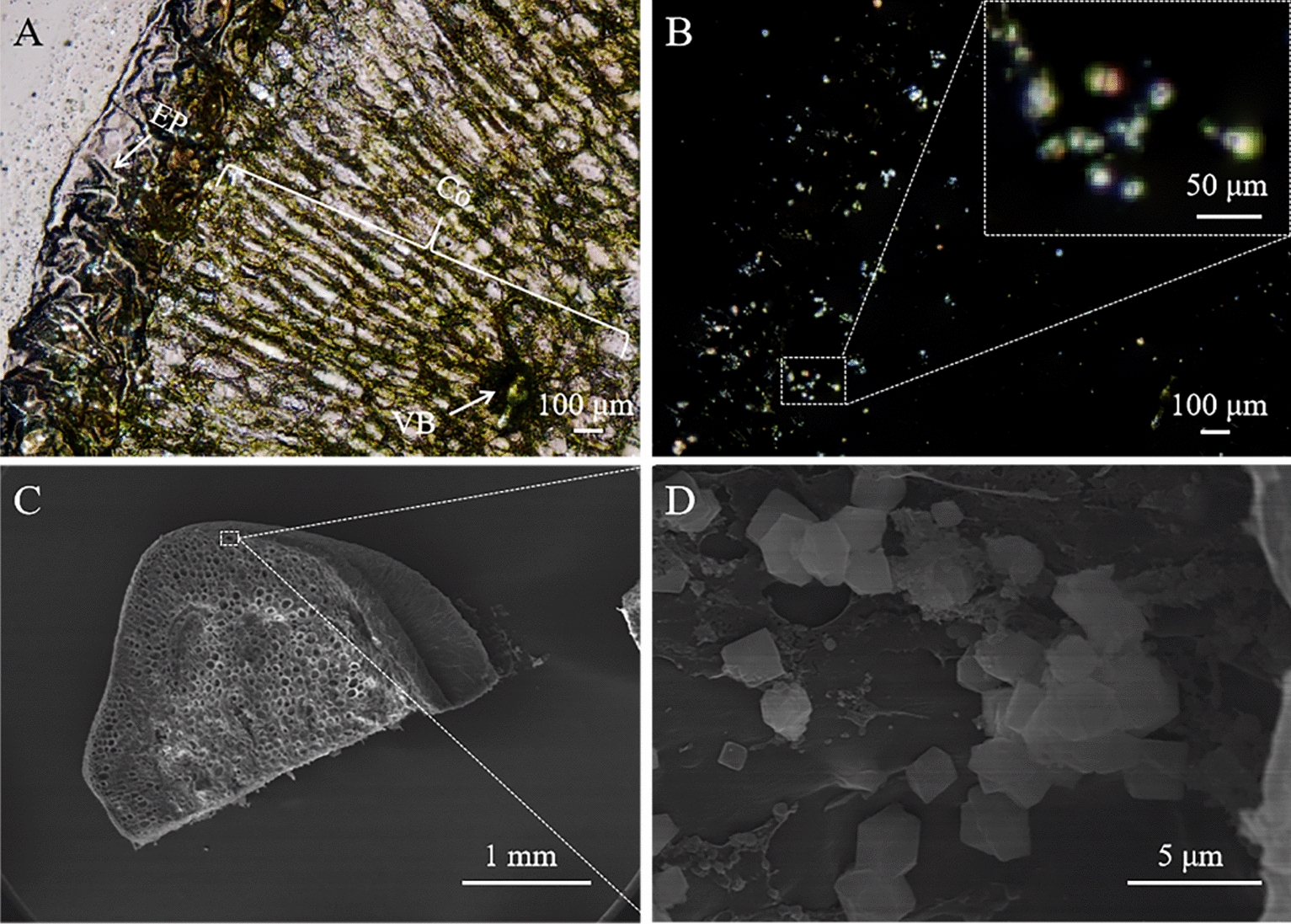
Polarizing microscopy images of a cross section of the tubercle under the modes of bright (**A**) and dark field (**B**), showing small-sized crystals distribution, and a magnified SEM image (**D**) from a white rectangle in a cross section of the tubercle (**C**), displaying the morphology of the small-sized crystals. (EP, epidermis; Co, cortex; VB, vascular bundles)

It was observed that only large-sized (> 40 μm) crystals with a prism shape through the mechanical blending (Fig. 4A and C). However, it should be noted that using the differential centrifugation after the mechanical blending can obtain massive small-sized (≤ 20 μm) crystals with tetrahedral or spherical shapes (Fig. 4B and D), which were consistent with the crystals being observed in epidermal and cortical cells from the tubercles of *Mammillaria schumannii* (Fig. 2)*.*

### Structural characteristics (EDS and Raman spectroscopy) of tetrahedral or spherical CaOx crystals

**Fig. 5:**
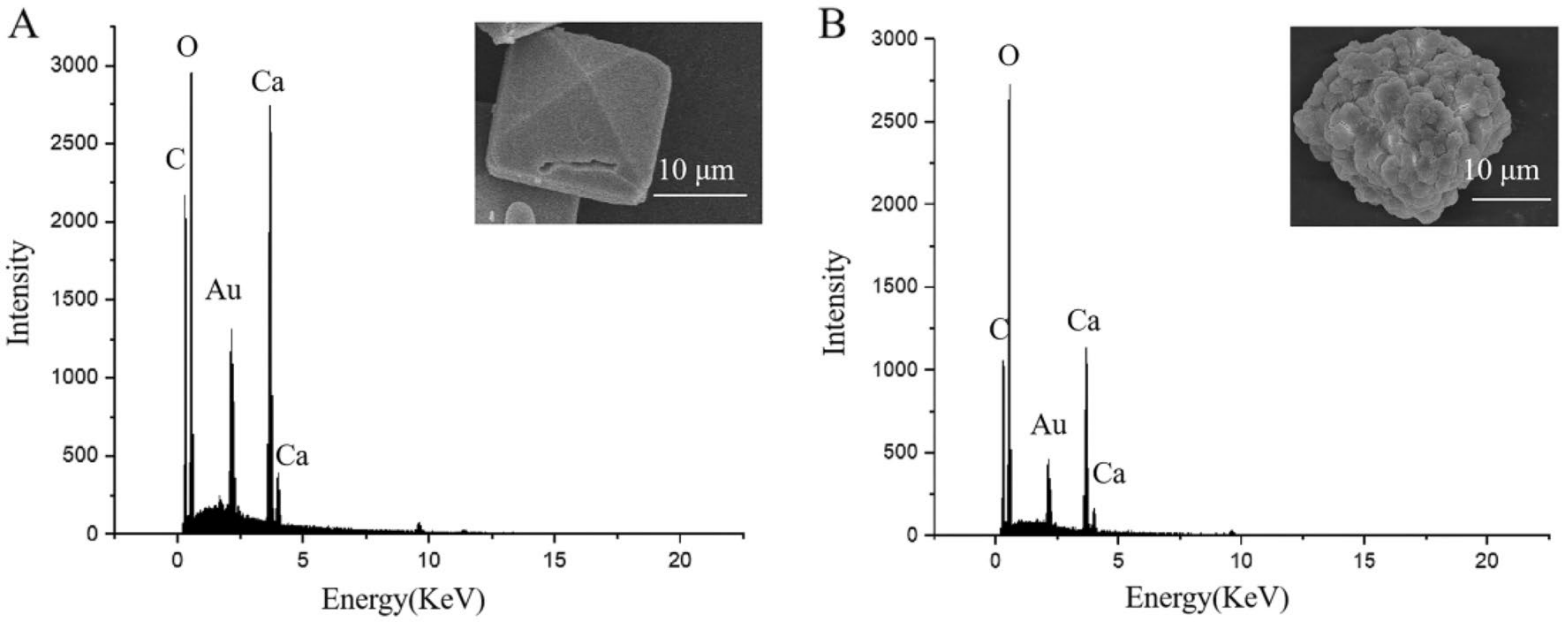
EDS analysis of extracted crystals with tetrahedral (**A**) and spherical shapes (**B**)

**Fig. 6:**
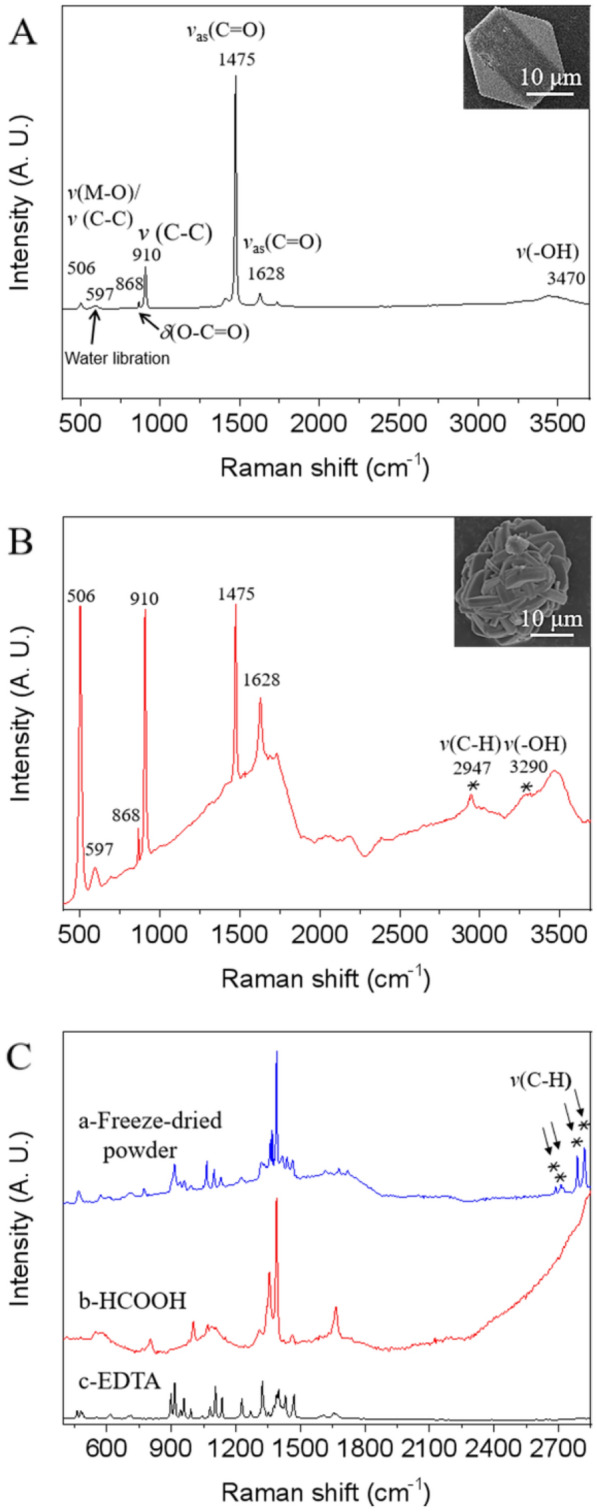
Raman spectroscopy analysis of isolated tetrahedral (**A**) and spherical (**B**) crystals, and the freeze-dried power organic matrix included in spherical durses extracted by HCOOH and EDTA(**C**)

The Energy Dispersive Spectroscopy (EDS) analysis was performed on the crystals isolated by differential centrifugation after mechanical crushing. Tetrahedral or spherical crystals were identified as Ca-C-O rich phases by representative element analysis (Fig. 5A and B). Further, characteristic Raman spectroscopy bands at 1475 and 1628 cm^−1^ were present in tetrahedral and spherical crystals, which were asymmetric stretching vibrational peaks of C = O (Fig. 6A and B) [27]. These two peaks are the characteristic peaks of CaC_2_O_4_.2H_2_O. Moreover, the CaC_2_O_4_.2H_2_O fingerprint peaks of 506, 597, 868, and 910 cm^−1^ (the vibrational peak of M–O、C–C、O-C = O) and the -OH vibrational peak of H_2_O at 3470 cm^−1^ also appeared in extracted crystals with tetrahedral and spherical shapes [28–30]. It is worth noting that two abnormal Raman spectroscopy bands (2947 cm^−1^ and 3290 cm^−1^) in the spherical CaC_2_O_4_.2H_2_O, were the stretching vibrational peaks of C-H and -OH bonds, indicating that the spherical druse crystals may contain organic matrix [31].

The organic matrix was further extracted from the spherical druses in order to identify its structure. The spherical crystals were first dissolved in 0.5 M EDTA to remove the surface organic matrix, and then the remaining materials were further treated with 78% formic acid to dissolve the occluded organic matrix. And Raman spectroscopy was used to examine the resultant supernatant that contained the organic matrix that had been liberated. The presence of obvious Raman spectroscopy peaks at 2650, 2720, 2770, and 2958 cm^−1^ (the stretching vibrational peak of C-H) (Fig. 6C), indicated that the occluded organic matrix in spherical CaOx can be polysaccharide, flavone, or lipid compounds [32, 33].

## Discussion

Here we used a highlightedly improved method to effectively isolate small-sized CaOx crystals dominating in the epidermal and cortical cells from tubercles of *Mammillaria schumannii*, which extremely tended to adhere plant tissues or suspend in isolation solution. Firstly, only larger CaOx crystals with a size of ≥ 40 µm were usually released through mechanical crushing of plant tissues (Fig. 4A and C). In contrast, we observed small-sized CaOx Crystals predominated in the cortical cells of the photosynthetic organ of cactus in our research (Fig. 2), which were mostly left out through their adhesion to tissue or suspension in solution. Here we used differential centrifugation after mechanical crushing to effectively isolate small-sized CaOx crystals (≤ 20 µm) from the tubercles of *Mammillaria schumannii* (Fig. 4B and D). On the one hand, gradually increasing the speed of centrifugation can settle very tiny floating materials, including crystals and plant tissues as far as possible. On the other hand, higher speeds of centrifugation can pick up those crystals because they are in close contact with plant tissues. Comparatively speaking to other extracted processes using protoplast isolation solution, the method of differential centrifugation after mechanical crushing can detach small-sized CaOx crystals from tissue pieces and has the advantages of convenience and time-saving in contrast to other extracted processes with protoplast isolation solution [2–4]. In addition, the added reagents can change the shape of CaOx crystals such as isopropanol (Additional file 1: Figure S3).

In our research, the small-sized crystals with tetrahedral or spherical morphologies that were isolated were identified as mainly CaC_2_O_4_·2H_2_O, and organic matrix was confirmed to get involved in the formation of spherical CaOx crystals. A key feature of calcium oxalate crystallization in plants is species-specific crystal morphology [5]. Exactly, Hartl et al. [34]. observed that raphides commonly existed in Hylocereus and Selenicereus species. In addition, it was clearly demonstrated that the direct relationship established between a given Cactaceae species and a definite calcium oxalate biomineral seems to be a useful tool for plant identification and chemotaxonomy [7]. For example, whewellite (CaC_2_O_4_.H_2_O) was found in seven different species of the Opuntioideae subfamily, and weddellite (CaC_2_O_4._2H_2_O) was found in an equal number of species of the Cereoideae subfamily. Within crystal-forming cells, crystal chambers provide compartments bounded by a biological membrane, separating the crystallization space from the vacuolar sap. In this way, they allow regulation of physical factors such as pH, water, and ion composition and concentration inside the compartment. Exactly, whewellite is preponderantly formed in a solution with a high Ox concentration facing a relatively low Ca concentration, whereas weddellite is almost exclusively in a solution with a high Ca concentration facing a normal or slightly augmented Ox concentration [10]. Moreover, weddellite peaked in the pH range of 5–6.5. In our research, the morphology of CaOx crystals in *Mammillaria schumannii* were tetrahedral and spherical, and these two kinds of crystals were CaC_2_O_4_.2H_2_O by analysis of the combined SEM–EDS and Raman spectroscopy. For weddellite are usually tetrahedral, spherical crystals of CaC_2_O_4_.2H_2_O can be the inclusion of a diversity of macromolecules. For example, the significant change in shape of CaC_2_O_4_ crystals from prism to spherality after superior occlusion of vesicles with steric stabilizer chains comprising anionic carboxylate groups leads to improved performance [11, 35]. Exactly, the presence of Raman spectroscopy bands of C-H (2650, 2720, 2770, and 2958 cm^−1^) in our research (Fig. 5), indicating some organic matrix, possibly polysaccharides, flavonoids, or lipids existing in spherical druses. And the shape of CaC_2_O_4_.2H_2_O formed in the crystal chamber should depend significantly on this organic matrix.

This highlightedly improved method for isolating and characterizing biomineral crystals can be helpful to understand how CaOx crystals in cacti function against harsh environments such as strong light, high and cold temperature, and aridity. We discovered that the small-sized CaOx crystals were mainly distributed in the epidermis and cortical (parenchymal) cells (Fig. 2), while the large-sized crystals were mainly distributed in the spongy shaped parenchyma cells, near the vascular bundles (Additional file 1: Figure S3). The former usually has a function in the maintenance and photoprotection of the photosynthetic apparatus, and the latter aims to excrete the excessive calcium. [4, 5, 36, 37]. We hypothesize that this distribution approach may mediate light collection efficiency in mesophyll cells for carrying out photosynthesis in cacti (Additional file 1: Figure S4) since small-sized CaOx crystals are almost everywhere in epidermis and cortical cells (Fig. 2) [12]. This function could be closely related to the morphological and structural characteristics of small-sized CaOx crystals. But the molecular mechanism behind it still remains vague. In the follow-up study, physical, chemical, and biological methods should be used to detect the light scattering of CaOx crystals in these cells.

## Conclusions

Here we used a highlightedly improved method to effectively isolate small-sized CaOx crystals dominating in the epidermal and cortical cells from tubercles of *Mammillaria schumannii*, which extremely tended to adhere plant tissues or suspend in isolation solution. And then we further clarified the organic matrix getting involved in the formation of CaOx crystals. This improved method for isolating and characterizing biomineral crystals can be helpful to understand how CaOx crystals in cacti function against harsh environments such as strong light, high and cold temperature, and aridity.

## Material and methods

### Plant material

Cacti *Mammillaria schumannii* were collected from the Department of Life Science and Technology (Fig. 1). The lateral roots of the plants were removed to avoid any invisible injuries or fungal infections. The pruned cacti were kept in a cool and ventilated place for a week to have the wounds completely dried, and they were reproduced vegetatively in square plastic pots (7 × 5 × 7.3 cm). Using a basalt grain/peat soil mixture (7:3) and maintained in natural conditions.

### CaOx crystals isolation from tubercles of *Mammillaria schumannii*

Mechanical blending to isolate large-sized crystals: The tubercles of *Mammillaria schumannii* were cut off and transferred to a blender containing 100 mL of distilled water (Xiao Xiong, LLJ-CO4L5). Check the crushing status every 10 s, repeating the crushing process for 3–5 min until some crystals can be observed at the bottom of the crusher. The homogenate was filtered once with a 50 mesh filter screen, and then filtered once with a 250 mesh filter screen. Stir the homogenate with a pipette to resuspend the precipitates and immediately collect the white precipitates that settled down first; thus, the large-sized crystals can be isolated. Continually wash the crystals with distilled water until all tissue fragments are removed, and finally add anhydrous ethanol for washing.

Differential centrifugation after mechanical blending: The blending and filtering processes are the same as above. After being filtered with a 250 mesh filter, collect all the pre-paid homogenate in the beaker with a pipette and centrifuge 3 times for 3–5 min at 10,000 rpm, 11,000 rpm, and 12,000 rpm, respectively. Every time after centrifugation, remove the part of supernatant and the tissue fragments deposited at the bottom of the tube, then add some distilled water to resuspend the precipitates. Finally, wash the crystals with absolute alcohol, centrifuge again at 12,000 rpm for 1 min. Pure, small-sized crystals can be obtained after removing the supernatant and air-drying.

### Morphological and structural characteristics of in-situ or isolated CaOx crystals from tubercles of *Mammillaria schumannii*

*Polarized light microscopy* The fresh tubercles were free-hand sectioned to observe the anatomical structure of *Mammillaria schumannii* and distribution of CaOx crystals. Firstly, the samples were washed with distilled water, and cleaned with absorbent paper. The fresh tubercles were cut with a sharp blade as quickly as possible, and the sections were placed on the slides and observed and photographed with an Olympus BX51 polarized light microscope equipped with an Olympus DP70 digital camera.

*SEM–EDS* The isolated crystals or dried cross sections of tubercles were mounted on an aluminum stub with a double-sided carbon tape, and were analyzed by the combined SEM–EDS (HITACHI, SU8010; EDAX, Octane Plus).

***Raman spectroscopy*** (HORIBA, LabRAM HR Evolution): Before sample measurement, the monochromator of the spectrometer was calibrated with Raman spectroscopy scattering rays generated by a silicon wafer (520.7 cm^−1^). And the collected CaOx crystals were placed on the slide, waiting to be measured, scanning for 60 s. The entire scanning range (200–4000 cm^−1^) of the subtracted spectra was monitored for the proper solvent subtraction, the resolution was 2 cm^−1^. The spectra were collected along 120 cm^−1^· min^−1^, and the data was recorded every 1 cm^−1^. Raman spectroscopy signals were collected in the backscatter direction, and at least 5 different sample points were selected for testing and recording.

### The extraction of organic matrix from the CaOx crystals and their chemical characteristics

(1) Boil the collected crystals in the 2 mL 10% SDS solution for 10 min and repeat 5 times. Then treat the liquid with acetone (4 ℃, 24 h) to remove all the superficial organic matter.

(2) The refined crystals were dissolved with 0.5 M EDTA (PH = 9.4) at 4 ℃. The EDTA should be replaced with fresh one every day. About one week later, the volume of solids decreased by 90%—95% (w/v). The residues were collected and desalted by three rounds of dialysis against MilliQ-H_2_O using Spectra/Por^®^ 7 (Spectrum Laboratories, Inc.) dialysis tubing with a molecular weight of cutoff of 3000 Da, centrifuged and condensed at 3000–4000 *g* for 30 min. Since in this case the active macromolecules were retained in the dialysis tubing, the larger pore size membrane was preferred for rapid desalting. The insoluble matter was completely washed with deionized water and extracted with 78% formic acid (v/v) overnight at room temperature. The solution was clarified with centrifuge at 10,000 rpm for 10 min. The supernatant containing formic acid soluble organic matrix was freeze dried (Christ, Beta 2–8 LD plus) and the target organic matrix can be obtained.

(3) The extracted organic matrix was characterized with a laser confocal micro-Raman spectrometer (HORIBA, LabRAM HR Evolution).

### Statistical analysis

Process the data with Microsoft excel 2010. Conduct the statistics and analysis with Image J. Chart with Origin Pro 9.8.

### Supplementary Information

**Additional file 1: Figure S1.** Polarized microscopy images of a cross section of the tubercle under the modes of bright and dark field, showing large-sized crystals distribution. **Figure S2.** The process of collecting CaOx crystals from tubercles of* Mammillaria schumannii* through the method of differential centrifugation after mechanical blending. **Figure S3.** The morphology of isolated CaOx crytals can be changed by isopropanol. **Figure S4.** Schematic illustration of possible function for small-sized CaOx crystals with distribution in epidermal and cortical cells of tubercles of Cacti* Mammillaria schumannii.*

## Data Availability

The datasets used in this study is available from the corresponding author on reasonable request.
