# Supplementary material for: A highlightedly improved method for isolating and characterizing calcium oxalate crystals from tubercles of Mammillaria schumannii
Source: Plant Methods. 2023 Nov 27;19:135. doi: 10.1186/s13007-023-01110-1 (PMC10680252; doi:10.1186/s13007-023-01110-1)
Supplement: Supplementary file 1 — Additional file 1: Figure S1. Polarized microscopy images of a cross section of the tubercle under the modes of bright and dark field, showing large-sized crystals distribution. Figure S2. The process of collecting CaOx crystals from tubercles of Mammillaria schumannii through the method of differential centrifugation after mechanical blending. Figure S3. The morphology of isolated CaOx crytals can be changed by isopropanol. Figure S4. Schematic illustration of possible function for small-sized CaOx crystals with distribution in epidermal and cortical cells of tubercles of Cacti Mammillaria schumannii. [file 13007_2023_1110_MOESM1_ESM.docx]

**Supplementary Information for A highlightedly improved method for isolating and characterizing calcium oxalate crystals from *Mammillaria schumannii***

**S1**


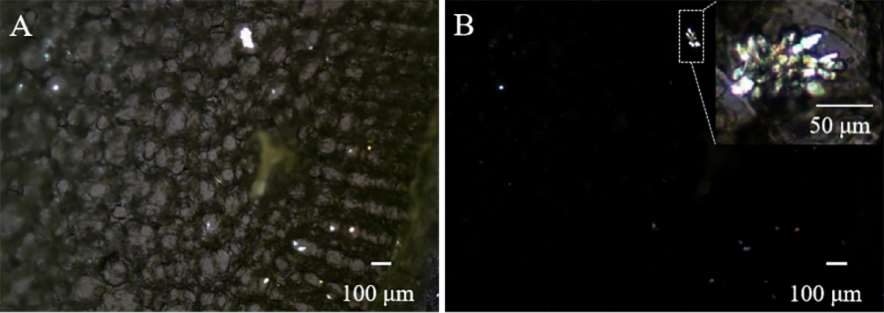


**Figure S1.** Polarizing microscopy images of a cross section of the tubercle under the modes of bright (**A**) and dark field (**B**), showing large-sized crystals distribution.

**S2**


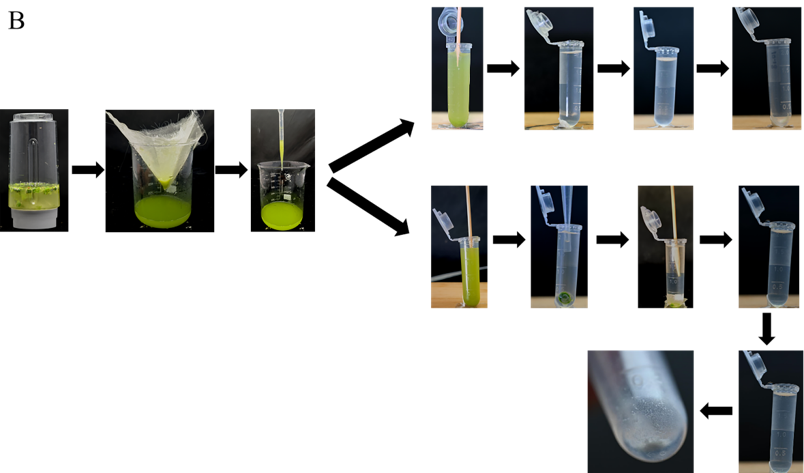


**Figure S2.** The process of collecting CaOx crystal from tubercles of *Mammillaria schumannii* through the method of differential centrifugation after the mechanical blending.

**S3**


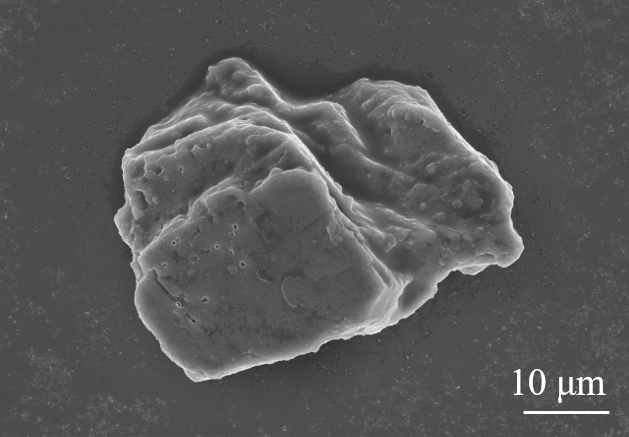


**Figure S3.** The morphology of extracted CaOx crytals can be changed by isopropylketone.

**S4**


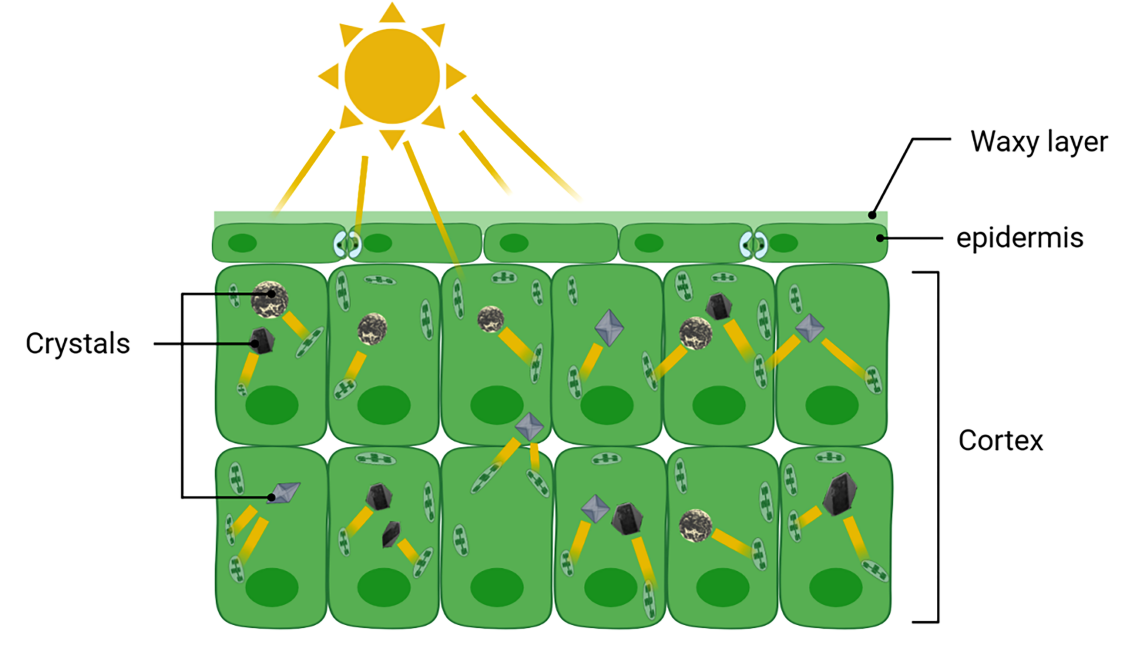


**Figure S4.** Schematic illustration of possible function for small-sized CaOx crystals with distribution in epidermal and cortical cells of tubercles of Cacti *Mammillaria schumannii.* When plants exposed to the sun, CaOx crystals in cells can capture light and deflect it to neighboring chloroplasts, thereby increasing photosynthesis.
